# A Comprehensive Investigation Into the Outcomes of Descemet’s Stripping Endothelial Keratoplasty (DSEK) as a Treatment for Corneal Endothelial Disorders

**DOI:** 10.7759/cureus.46076

**Published:** 2023-09-27

**Authors:** Amritha Tilak, Jayashree Dora, Kanhei C Tudu, Gopeswari Hota, Sharmistha Behera

**Affiliations:** 1 Department of Ophthalmology, Veer Surendra Sai Institute of Medical Sciences and Research, Burla, IND

**Keywords:** complications, visual recovery, surgical outcomes, corneal endothelial disorder, descemet's stripping endothelial keratoplasty

## Abstract

Background and objectives: Descemet's stripping endothelial keratoplasty (DSEK) has emerged as the preferred method for posterior lamellar keratoplasty, as it enables the replacement of the compromised host endothelium with a viable donor lamella. The objective of this study was to assess the impact of DSEK on surgical outcomes and visual acuity.

Methods: The research was carried out from November 2019 to October 2021, encompassing a sample of 18 patients (18 eyes) who satisfied the inclusion criteria for DSEK. The pre-operative evaluation was performed once before the surgery, while post-operative evaluations were conducted at one, three, and six months after the surgical intervention. The main variables assessed in this study encompassed demographic characteristics, visual acuity, surgical techniques, and surgical complications employed during the surgical procedure. The collected data were statistically analyzed using IBM SPSS software version 21 (IBM Corp., Armonk, NY, USA).

Results: Patients in the study ranged in age from 25 to 70, with a mean age of 53.16 ± 14.19 years. The participants were 61% male and 39% female. The main reasons for DSEK use were pseudophakic bullous keratopathy (61%) and post-penetrating keratoplasty (PK) graft failure (17%). The other indications were aphakic bullous keratopathy (11%), bullous keratopathy with cataracts (5%), and Fuchs's endothelial dystrophy (5%). The study included 18 eyes: 14 eyes underwent DSEK, two underwent DSEK with small-incision cataract surgery (SICS) and posterior chamber intraocular lens (PCIOL) implantation, and two underwent DSEK with scleral-fixated FIL. A significant improvement in best-corrected visual acuity (BCVA) was observed at six-month follow-up (0.73 ± 0.37 vs. 1.73 ± 0.59 logMAR pre-operatively). During donor cornea dissection, buttonholing was the main concern, occurring 11% of the time. Descemet's perforation and donor preparation thickness variations were also observed. Reverse donor unfolding, incomplete DM stripping, and donor button displacement were quickly addressed and managed. Post-operative graft failure occurred in 22% of cases, while acute graft rejection occurred in 11%. Graft dislocation, pupillary block, and secondary glaucoma each had a 5% prevalence.

Conclusion: Descemet's stripping endothelial keratoplasty is a safe and effective treatment for corneal endothelial disorders, particularly in cases where scarring is not present. Surgical skills are essential to achieving the desired results. Descemet's stripping endothelial keratoplasty is favored over conventional keratoplasty for endothelial dysfunction due to its technical advantages and manageable risks. Our research demonstrates a significant improvement in visual acuity through DSEK. Despite manageable post-operative complications, it is vital to educate patients and medical professionals on surgical complexities. Descemet's stripping endothelial keratoplasty appears promising for the treatment of endothelial disorders, but its long-term implications must be studied.

## Introduction

Corneal blindness is a significant public health concern in developing countries, ranking second only to cataracts, uncorrected refractive errors, and glaucoma [1]. The World Health Organization (WHO) claims that corneal conditions are a significant cause of vision loss [1]. According to the findings of the national blindness and visual impairment survey in India for the period of 2015-2019, corneal opacities have been identified as the primary cause of blindness, particularly among individuals below the age of 49 [2]. Ocular trauma and corneal ulceration are thought to be the main causes of corneal blindness in developing countries, together accounting for about 90% of cases [3, 4].

Corneal transplantation is the main therapeutic strategy for treating corneal opacity. Significant surgical progress has been made in the field of keratoplasty, which has fundamentally altered how corneal diseases are treated. As effective alternatives to penetrating keratoplasty (PK) for the treatment of corneal pathologies, lamellar techniques have grown significantly in popularity. Lamellar keratoplasty (LK) is a surgical procedure used only to replace compromised corneal tissue [5, 6, 7]. Endothelial keratoplasty (EK) was first introduced as a surgical procedure by Melles et al. [8], with a focus on posterior lamellar keratoplasty (PLK), which seeks to replace the posterior corneal layers and endothelium. In cases of bullous keratopathy and Fuchs' corneal dystrophy, it has been demonstrated that the edema can be successfully reduced by replacing an unhealthy host endothelium with a healthy donor endothelium [9]. Smooth surface topography, stable corneal power, excellent optical quality, tectonic stability, and patient safety are the goals of endothelial transplantation [10].

Through its barrier and pump functions, the corneal endothelium is a key component in preserving the transparency of the cornea. Endothelial dysfunction leads to corneal edema and vision loss. Numerous conditions, such as pseudophakic or aphakic bullous keratopathy, Fuchs' endothelial corneal dystrophy, posterior polymorphous dystrophy, trauma-related conditions, and other hereditary disorders, can result in corneal decompensation [11]. As the predominant primary cause of corneal endothelial dysfunction globally, Fuchs' endothelial corneal dystrophy is widely acknowledged, while pseudophakic or aphakic bullous keratopathy is recognized as the predominant secondary cause [11].

Due to its technical benefits, manageable side effects, and improved visual rehabilitation, Descemet's stripping endothelial keratoplasty (DSEK) has grown significantly in popularity as a treatment for corneal endothelial dysfunction [12]. With fewer sutures, improved tectonic stability, no early post-operative nerve supply, and a quicker visual recovery, DSEK outperforms PK in several ways [12]. Descemet's stripping endothelial keratoplasty can be performed using manual, automated, or femtosecond laser-assisted techniques, and cost-effectiveness and regional specificity are key factors in selecting this technique [13].

Compared to penetrating keratoplasty, DSEK has several benefits, and it has been discovered to significantly improve tectonic stability and reduce astigmatism. Refractive stability and visual acuity recovery are expedited [14]. The use of DSEK has been shown to improve endothelial disease management outcomes; however, it's important to remember that dislocation can happen due to poor donor endothelial adherence or trauma [14].

The treatment of corneal endothelial dysfunction has undergone a significant change since the introduction of DSEK. Compared to PK, this technique is more effective and safer in terms of visual results and complications. Due to its inherent advantages over standard procedures, DSEK has the potential to improve patient outcomes in cases of corneal endothelial dysfunction. The purpose of the current study is to better understand the visual outcomes, indications, intra-operative and post-operative complications, and follow-up uncorrected visual acuity (UCVA) and best-corrected visual acuity (BCVA) of DSEK.

## Materials and methods

Ethical approval and study initiation

The study was conducted after review and written approval by the Veer Surendra Sai Institute of Medical Science and Research (VIMSAR) Institutional Review Board, specifically the VIMSAR Institute of Research and Ethics Committee (VIREC) (approval no: 19212/Dt-30.11.19/IST-225/19).

Study design and participant selection

The study was carried out at the Veer Surendra Sai (VSS) Institute of Medical Science and Research in Burla, Sambalpur, Odisha, India, between November 2019 and October 2021, within the Department of Ophthalmology, encompassing both the outpatient department (OPD) and the operating room (OT). All patients who underwent DSEK both before and after were included in the study.

Patient enrollment and sample size estimation

The sample size estimation was based on a 0.05 α error and a 0.1 β error. The sample size was 23 after using a sample size calculator and accounting for a 36% difference in proportion (logMAR 0.40-0.79) from a prior study by Kadam and Bhalerao [15]. Nevertheless, only 18 patients were successfully selected for the study because of the COVID-19 pandemic's restrictions. Sequential sampling was used in the patient selection technique. 

Subject selection

Inclusion Criteria

The present study included patients with a range of corneal conditions based on specific inclusion criteria. Clinical indicators like thickening of the cornea and guttae, as well as symptoms like decreased morning vision, glare, and blurred vision, helped to diagnose Fuchs' endothelial dystrophy (FED). Congenital hereditary endothelial dystrophy (CHED) was thought to be the cause if both of a child's corneas were cloudy from an early age, there was a family history of CHED, there were changes in the corneas, and genetic testing confirmed specific mutations. Pseudophakic or aphakic bullous keratopathy was identified when individuals who had undergone cataract surgery (pseudophakic) or lens removal (aphakic) experienced corneal swelling and fluid accumulation in the affected eye. Patients with failed keratoplasty due to endothelial rejection were included, focusing on the involvement of the endothelial layer of the donor cornea. Additionally, cases of post-infective endotheliitis were considered based on patients' histories of prior eye infections and clinical signs like corneal inflammation and edema.

Exclusion Criteria

Patients with corneal stromal scarring, uncontrolled glaucoma, an irregular and deformed anterior chamber, gross peripheral anterior synechia (PAS), and gross posterior segment pathology were excluded from the study, according to the B-scan ultrasound results. A thorough history-taking process and a slit-lamp examination were used to determine the disease's diagnosis. All patients provided written informed consent confirming their understanding of potential surgical outcomes and complications in the language of their choice. The follow-up procedures related to the study were fully explained to the patients.

Specular microscopy

Specular microscopy was performed using an ophthalmic device, with patients prepared through topical anesthetic eye drops and comfortable seating. The instrument was calibrated, and multiple high-resolution images of the corneal endothelium were captured. The subsequent analysis assessed the density and morphology of endothelial cells. The safety and comfort of the patient were top priorities throughout the procedure. For a comprehensive evaluation, specular microscopy data were combined with other clinical observations, especially when a condition such as Fuchs' endothelial dystrophy (FED) was present.

Surgical procedures and the preparation of donor tissue

The Barron artificial anterior chamber was used to mount the donor cornea and scleral rim, with the endothelial side facing down. After mounting, a guarded blade was used to make an initial 5 mm corneal incision at the limbus. The cornea's center was then marked. A lamellar corneal dissector was then used to perform manual lamellar dissection at about two-thirds depth. The endothelial side of the donor tissue was then placed facing up on a Teflon block. An endothelial punch was used to trephine a donor lenticule of the right size.

Corneal marking and preparation of the recipient’s bed

On the corneal epithelial surface, a circular template mark with a diameter of 7.5, 8.0, 8.5, or 9.0 mm was used as a guide for Descemet stripping. After making a conjunctival flap and using wet field cautery, a 5.0 to 5.5 mm sclero-corneal tunnel was made. At 10 and two o'clock, two 1-mm side ports were made so that the donor lenticule could be moved and opened more easily. Then, 1% sodium hyaluronate, a cohesive viscoelastic agent, was injected through the side port. With a 2.8-mm angular keratome, the anterior chamber was opened, and a Descemet score, or circular dissection of the Descemet membrane, was done with a turned-around Sinsky hook. With the help of the hook, the Descemet membrane was taken off completely, getting rid of the diseased tissue. To ensure a clear view of the anterior chamber, it was thoroughly rinsed with a balanced salt solution (BSS).

Application of viscoelastics and entry into the anterior chamber

Donor Lenticule Transplantation

The endothelial side of the posterior lamellar donor lenticule was removed using a 7.5, 8.0, 8.5, or 9.0 mm single-use trephine. The recipient's cornea was then covered with the endothelial side of the lenticule. The lenticule was protected by a thin viscoelastic coating. The lenticule was gently guided into the anterior chamber through the tunnel with the aid of a sheet glide. A BSS was used to fill the anterior chamber and form a bubble after controlled air injection through a 30-gauge cannula. After the cornea was massaged with an iris repositor to center the lenticule, a clear "golden ring" could be seen around its edge. The interface fluid was removed by gently massaging and stroking the corneal epithelial surface. The primary wound was stitched with 10-0 nylon interrupted sutures following the rehydration of the side ports. The anterior chamber was infused with air for a predetermined period. The patient was instructed to stay in the supine position for four to six hours.

Post-operative follow-up and management

Prednisolone acetate (1%), a topical medication, was applied six times daily. Unlike PK cases, this regimen could be tapered gradually and stopped earlier. Broad-spectrum antibiotics, including gatifloxacin, were topically applied four times daily for a period of three to six months. In our study, the number of sutures applied varied based on factors such as wound size, location, surgical type, and surgeon preference. For small wounds, a single suture sufficed, whereas larger incisions or complex surgeries required multiple sutures. Sutures were removed when they got loose, got infected, or broke. Patients were kept in the hospital for at least five days after surgery. Subsequent follow-up appointments were scheduled for intervals of one month, three months, and six months, and the intraocular pressure (IOP), UCVA, and BCVA were all evaluated at each follow-up appointment.

Optical evaluation and research variables

Tonometry using a non-contact tonometer or digital tension measurement was performed before and after DSEK, along with UCVA, BCVA, slit-lamp examinations, and tonometry. Age (in years), gender (male/female), the recipient's lens status (pseudophakic/phakic/aphakic), the diagnostic information that led to the surgical procedure, the surgical technique, pre-and post-operative IOP (measured in mm Hg), UCVA, and BCVA, as well as intra- and post-operative complications, were among the many factors that were meticulously recorded.

Statistical analysis

The collected data were subjected to a comprehensive statistical analysis using the IBM SPSS software version 21 (IBM Corp., Armonk, NY). This analysis aimed to extract valuable insights from the study's findings and draw dependable conclusions.

## Results

The study examined the efficacy of DSEK in treating patients with corneal endothelium disorders. The study involved a cohort of 18 patients and one eye for each patient, with an average age of 53.16 ± 14.19 years, ranging from 25 to 70 years, used for the analysis (Table 1).

**Table 1:** A comprehensive summary of the demographic parameters employed in the present study DSEK: Descemet's stripping endothelial keratoplasty; SICS: small incision cataract surgery; PCIOL: posterior chamber intraocular lens; SFIOL: sulcus-fixated intraocular lens

Characteristics		Value
Eyes (no.)		18
Age (years)		
	Mean ± SD	53.16 ± 14.19
	Range	25-70
Age groups (years/no.)		25-40 (3), 41-55 (6), 56-70 (9)
Patients (no./%)		
	Male	11 (61)
	Female	7 (39)
Lens status (baseline; no./%)		
	Pseudophakic (posterior chamber intraocular lens; PCIOL)	11 (61)
	Phakic	5 (28)
	Aphakic	2 (11)
Indication for transplant (no./%)		
	Pseudophakic bullous keratopathy	11 (61)
	Failed penetrating keratoplasty	3 (17)
	Aphakic bullous keratopathy	2 (11)
	Bullous keratopathy with cataract	1 (5)
	Fuchs endothelial dystrophy (FED)	1 (5)
Surgical technique (no./%)		
	DSEK	14 (78)
	SICS + PCIOL with DSEK	2 (11)
	DSEK with SFIOL	2 (11)

In this study, a male-to-female ratio of 1.57:1 was found, indicating a slightly higher prevalence of male patients within the analyzed cohort. Out of a total of 18 patients, 11 patients (61%) were male, while seven patients (39%) were female (Table 1). According to the findings, pseudophakic eyes predominated, making up 61% of the sample (11 eyes) out of the total sample size of 18 eyes (Table 1). The standardized surgical procedure was used to ensure consistent implantation of posterior chamber intraocular lenses (PCIOL) in the affected ocular organs. Furthermore, the successful preservation of the natural crystalline lens was demonstrated in 28% of the ocular organs (five ocular organs), maintaining their phakic status. It's important to note that two eyes, or 11% of the total, had an aphakic condition (Table 1).

Pseudophakic bullous keratopathy (PBK) was identified as the predominant diagnosis, comprising 61% (11 eyes), failed penetrating keratoplasty (PK) was observed in 17% (three eyes), and aphakic bullous keratopathy was present in 11% (two eyes) out of a total of 18 eyes (Table 1). A single patient presented with bullous keratopathy and cataracts in the other eye after experiencing unexplained corneal swelling following cataract surgery in one eye and DSEK elsewhere.

Descemet's stripping endothelial keratoplasty was performed on 14 out of 18 eyes or 78% of the total cases. Eleven eyes had PBK, three eyes had previously failed PK grafts and one eye had FED (Table 1). Two eyes underwent small-incision cataract surgery (SICS) + PCIOL combined with DSEK, one of which was diagnosed with bullous keratopathy, cataracts, and a history of failed PK. Moreover, DSEK with a sulcus-fixed intraocular lens (SFIOL) was performed on two aphakic eyes.

In this study, all 18 patients had normal pre-operative IOP, indicating the absence of high IOP as a risk factor (Table 2).

**Table 2:** Variations in intraocular pressure (IOP) throughout follow-up for up to six months after cataract surgery

Characteristics	Normal (16 mmHg)	Increased (25 mmHg)
Pre-operative	18	0
Immediate post-operative	17	1
1 month	17	1
3 months	18	0
6 months	18	0

No abnormal IOP levels were detected before cataract surgery, affirming the lack of IOP-related concerns. It's important to note that one patient experienced a pupillary block (an air bubble-related IOP increase of 25 mmHg) despite postoperative cycloplegia in the immediate postoperative period. This patient was immediately taken for the release of air bubbles, and the patient's IOP normalized, while the IOP of the remaining 17 patients remained normal. All patients confirmed that their IOP remained within normal ranges three and six months after surgery, which indicates that regular post-operative assessments consistently showed normal IOP readings within the range of 12 to 19 mmHg. These values suggest no sustained abnormal increases in IOP among the patients during the specified post-operative periods. These consistent results highlight the significance of post-cataract surgery IOP management, which is essential for enhancing the overall success and safety of the surgical intervention (Table 2).

Prior to cataract surgery, the UCVA was 1.73 ± 0.59 logMAR, with a range of 1.08 to 2.80 (Table 3), and follow-up assessments of visual acuity revealed a significant change.

**Table 3:** Uncorrected visual acuity (UCVA) and best-corrected visual acuity (BCVA) change up to six months following cataract surgery.

Follow-up period	No.	Mean ± SD (logMAR)	Min-Max	p-value
UCVA				0.001
Pre-operative	18	1.73 ± 0.59	1.08 – 2.80	
1 month	18	1.05 ± 0.30	0.50 – 1.78	
3 months	18	1.05 ± 0.33	0.40 – 1.80	
6 months	18	0.95 ± 0.34	0.50 – 1.78	
BCVA				
Pre-operative	18	1.73 ± 0.59	1.08 – 2.80	
6 months	18	0.73 ± 0.37	0.20 – 1.48	

A Friedman test analysis revealed that the UCVA values were 1.05 ± 0.30, 1.05 ± 0.33, and 0.95 ± 0.34 logMAR at one, three, and six months following surgery, respectively. For the corresponding time points, the UCVA range ranged from 0.50 to 1.78, 0.40 to 1.80, and 0.50 to 1.78. The pre-operative BCVA was 1.73 ± 0.59 logMAR, with a minimum and maximum range of 1.08 to 2.8, serving as a baseline for future improvements (Table 3). By the end of the six-month follow-up, the BCVA had reached 0.73 ± 0.37 logMAR and was statistically significant (p-value of 0.001, Wilcoxon signed-rank test). The transformation's range of values, from 0.20 to 1.48, demonstrated the variety of patient advancements.

Following a six-month examination using the Snellen chart, 50% (nine patients) were found to have a BCVA of 6/60 or worse, indicating severe visual impairment. This condition was unique to this group and was not observed in any other patients, highlighting the severity of their visual impairment (Table 4).

**Table 4:** Change in visual acuity from pre-operative to sixth-month follow-up: best-corrected visual acuity (BCVA) as per Snellen's chart

BCVA (n=18)	Pre-operative	6 months (%)
6\6	0	0 (0)
6\9	0	1 (6)
6\12	0	2 (11)
6\18	0	4 (22)
6\24	0	0 (0)
6\36	0	2 (11)
6\60 or worse	18	9 (50)

On the other hand, Snellen's chart shows that four of the patients, or 22%, had a BCVA of 6/18 or better. Two patients achieved BCVA measurements of 6/12 and 6/36, which is a remarkable improvement. These findings suggest a potential for vision improvement. This subset of results shows that there might be an improvement in vision after the surgery.

The incidence of intra-operative complications during corneal donor dissection and recipient procedures is shown in Table 5.

**Table 5:** Intra-operative complications during donor dissection and recipient procedure

During donor dissection	No. (%)
Buttonholing of the donor cornea	2 (11)
Excessive thick donor preparation (500 µm)	1 (5)
Descemet’s perforation of the donor cornea	1 (5)
Too thin donor preparation (200 µm)	1 (5)
During the recipient procedure	
Reverse unfolding of donor	1 (5)
Donor button came out of the anterior chamber	1 (5)
Incomplete stripping of the Descemet's membrane	1 (5)

Two patients (11% of cases) in this study had buttonholes in the corneal donor, indicating accidental perforations. In addition, one case (5%) exhibited excessively thick donor tissue, while another case (5%) exhibited tissue thinness, indicating differences in tissue dimensions. In two cases (5%) with buttonholes in the donor cornea, it's important to note that there was no concurrent Descemet's perforation in these instances. Importantly, these complications arose during procedures involving donor dissection. The donor cornea experienced reverse unfolding in a single case (5%), indicating a complication originating from the donor cornea within the recipient's eye (Table 5). A donor button was accidentally dislodged from the anterior chamber (AC) in another single case (5%), indicating inadvertent tissue displacement. Surgical removal of Descemet's membrane (DM) was observed to be incomplete in one additional case (5%). These complications of intra-operative surgery serve as surgical focal points, highlighting potential areas of vigilance during donor dissection and recipient procedures.

Table 6 summarizes post-operative complications resulting from the surgical interventions conducted. Graft failure emerged as the foremost issue, afflicting four patients (22%).

**Table 6:** Incidence of post-operative complications

Post-operative complications	No. (%)
Graft failure	4 (22)
Acute graft rejection	2 (11)
Graft dislocation	1 (5)
Pupillary block glaucoma (air-induced)	1 (5)
Secondary glaucoma (steroid-induced)	1 (5)

Graft failure was successfully managed through regrafting procedures for the affected patients. Acute graft rejection affected two patients (11%), whereas each complication of graft dislocation, pupillary block glaucoma triggered by air, and secondary glaucoma induced by steroids manifested in one patient (5%), respectively. Acute graft rejection cases responded well to prompt and appropriate medical interventions, leading to graft survival without further complications.

## Discussion

The DSEK technique is widely accepted as a valuable methodology in the field of ophthalmology for the treatment of endothelial dysfunction. This technique entails the deliberate substitution of the impaired endothelium, resulting in enhanced patient outcomes. The aim of this study was to evaluate the visual and surgical outcomes linked to DSEK as a therapeutic modality for endothelial dysfunction. The average age of the patients in this study was 53.16 ± 14.19 years, with 61% of the participants being male and 39% being female. Notably, Price and Price [16] and Rice et al. [17] reported significantly older average ages of 70 ± 12 years and 69 ± 11 years, respectively, in previous studies. This variation in average age illustrates a significant difference between the demographics of this study's participants and those of previous studies, highlighting the unique younger age distribution of this research cohort.

In our study, 61% of the eyes were pseudophakic, indicating that they had previously undergone cataract surgery with intraocular lens implantation. Furthermore, 28% of eyes retained their natural crystalline lenses (phakic), while 11% were aphakic, indicating that the natural lens was missing due to surgery or congenital factors. Most congenital issues are identified in infancy or childhood, but some may not become clinically significant until adulthood, especially between 25 and 70. Congenital heart defects, connective tissue disorders like Ehlers-Danlos syndrome, and genetic or metabolic conditions may not be detected until adulthood. Early diagnosis and medical evaluation are essential for treating these conditions. In comparison, the proportions in the study by Basak [18] were slightly different. Among 75 eyes, 24% were phakic, with the vast majority, 76%, being pseudophakic. Our study found differences in eye condition prevalence compared to Basak [17]. In our cohort of 18 eyes, 61% were pseudophakic, indicating cataract surgery with intraocular lens implantation; 28% retained their natural crystalline lenses; and 11% were aphakic, indicating surgery or congenital lens absence. Basak's study, which included 75 eyes, found 24% phakic and 76% pseudophakic. This variation shows how sample size affects eye condition distribution, as larger samples better represent population prevalence. Additionally, patient demographics, geographic regions, and study inclusion criteria may have contributed to the observed condition prevalence differences between the two studies. These differences emphasize the importance of study context when interpreting and comparing findings. Both studies provide valuable insights into the prevalence of eye conditions in their respective contexts. Our 18-eye cohort provides valuable data for a specific patient group, while Basak's larger sample size provides a broader view of condition prevalence. These differences help us understand eye conditions in different patient populations and emphasize the need for contextual interpretation in research comparisons.

In this study, pseudophakic bullous keratopathy was the primary indication that necessitated the implementation of DSEK and accounted for 61% of the observed cases. It's noteworthy that in 17% of the cases, DSEK was performed to establish this primary key indication, although these efforts ultimately did not succeed. Further analysis of the data showed that aphakic bullous keratopathy was present in 11% of the cases. A rare simultaneous occurrence of bullous keratopathy and cataracts was seen in 5% of cases [19]. Additionally, a prevalence rate of 5% for FED was discovered in the cohort under study. There is a significant congruence between these findings and those of Basak [18] and Price and Price [16]. Fuchs endothelial dystrophy was acknowledged in both earlier studies as a key indicator of DSEK. According to Basak [17], a significant percentage of eyes (70.7%) that underwent DSEK had pre-existing pseudophakic bullous keratopathy rather than pseudophakic bullous keratopathy developing due to the DSEK procedure.

Our study describes a rare case of bullous keratopathy in one eye after cataract surgery and cataracts in the other after DSEK. This case contrasts with Fukuoka et al. [20], who reported bullous keratopathy and malignant glaucoma in the same eye. Bates and Cheng [21] examined endothelial cell morphology before and after bullous keratopathy eye surgery, but our study differed. These differences highlight the uniqueness of our case and its potential to illuminate corneal surgery dynamics and outcomes, especially when complications occur in different eyes after different surgeries.

The visual acuity outcomes of the study provide valuable insights into the effects of surgical interventions on patients with corneal disorders. The subjects' UCVA was found to be significantly impaired prior to DSEK surgery, indicating the difficulties they faced. Subsequent assessments revealed consistent improvements in UCVA over time. Statistical analysis, such as the Friedman test, allowed for a comprehensive evaluation of post-surgery UCVA trends. Notably, UCVA values at one, three, and six months after surgery showed a significant improvement in visual acuity. The narrowing of UCVA ranges for corresponding time points suggests improved visual consistency. These findings highlight the efficacy of surgical interventions in improving visual acuity in people with corneal disorders.

In our study, the BCVA at six months improved significantly, measuring 0.73 ± 0.37 logMAR compared to the pre-operative BCVA of 1.73 ± 0.59 logMAR (p-value: 0.001). This positive trend is consistent with the findings of Basak [18], who discovered that approximately 80% of cases achieved a post-operative BCVA of 0.5 logMAR or better after three months. Similarly, after nine months, Lee et al. [13] reported BCVA values ranging from 0.2 to 0.5 logMAR. Koenig [22] reported a pre-operative BCVA of 0.7 logMAR, which improved to 0.3 logMAR six months after surgery. Additionally, Rice et al. [16] documented a pre-operative BCVA of 1.2 logMAR, which demonstrated a reduction to 0.48 logMAR in the post-operative evaluation. Patients who did not have complications had their optimal post-operative spectacle correction within six months, whereas PK patients typically had sutures removed after a year, followed by a final spectacle correction. These findings highlight the overall beneficial effect of surgical interventions on visual acuity.

Several complications occurred in the current study's donor cornea dissection, including 11% of cases of buttonholes and 5% of cases of Descemet's perforation. Additionally, we encountered specific challenges related to donor tissue characteristics, where 5% of cases exhibited increased viscosity, indicating thicker or more resistant tissue consistency, and in 5% of cases, issues related to decreased fluidity were observed, signifying reduced ease of tissue manipulation during preparation. These findings aligned with the Basak [18] study, underscoring their significance. Recipient procedure issues persisted, including reverse donor unfolding, incomplete DM stripping, and donor button dislodgement during air tamponade. This supports Basak's [18] findings and emphasizes the recipient phase's complexity. Dislocation rates showed interesting trends. Initially 18%, these gradually fell to 13%, which position changes and improved methods, such as vent placement, are credited for. In their most recent 64 cases, the detachment rate was reduced to 6%.

Variability was found when compared to earlier studies. Gorovoy [23] found a higher percentage of detachment than our study, 25% in 16 eyes. After making adjustments to their technique, Allan et al. [24] saw a drop in detachment rates from 18% to 4%. Mearza et al. [25] highlighted the varied results of DSEK by reporting 82% detachment in 11 cases. While Covert and Koenig [26] observed 14% detachment in 21 combined DSEK cases, Koenig and Covert [27] showed 35% detachment. Our study identified graft failure as the primary concern, affecting four patients (22%). This result aligns with previous findings reported by Price and Price [15], who, in a larger sample, observed a lower graft failure rate of 4%. However, it's significant to note that the graft failure rate in our study differs significantly from that in the study by Mearza et al. [21], which reported a noticeably higher graft failure rate of 45%. Covert and Koenig [22] and Koenig and Covert [23] found graft failure rates of 10% and 12%, respectively. We identified this complication in two patients (11%) with acute graft rejection. This is consistent with Allan et al. [20], who reported that 11% of their study also experienced acute graft rejection. Our study's findings on graft failure and acute graft rejection fall within the range of previously reported rates in the literature, demonstrating the variability in outcomes across different studies. It's essential to consider the study-specific factors, patient populations, and methodologies that may contribute to these variations in graft failure and acute graft rejection rates. In the published literature, secondary glaucoma rates ranged from 0% to 15%; Basak [18] reported an 11.8% rate that was primarily attributed to corticosteroid use. Our study's findings include the correlation between elevated IOP in one patient (5%) and the adverse effects of steroids. Furthermore, as documented in our study, we addressed an uncommon but important condition, pupillary block glaucoma, by partially removing an oversized air bubble. As a preventative measure against the pupillary block, Allan et al. [24] suggested using cycloplegics and a moveable air bubble.

The relatively small sample size of 18 eyes in the study group is the main limitation of our study. Due to the possibility of variability in larger-scale studies, care should be taken when interpreting the results. The global COVID-19 pandemic, which had a significant impact on the collection of ocular specimens and the execution of surgical interventions, served to exacerbate the restrictions. It is important to note that the study did not examine average refractive and keratometric astigmatisms, which could have offered insightful information. Additionally, people with significant iris defects, glaucoma filters, or visibly fixed and dilated pupils were not included in our study on DSEK. It is essential to extend the follow-up period in order to fully assess endothelial cell loss and graft survival after DSEK. Our study's relatively short six-month follow-up period underscores the necessity for future extensive research endeavors with prolonged follow-up periods. These studies will provide a comprehensive assessment of endothelial cell loss and graft survival following DSEK, providing a deeper insight into the procedure's long-term implications. Furthermore, our research scope will encompass various aspects, including patient-reported outcomes, quality-of-life assessments, and meticulous examination of refractive and keratometric astigmatisms. These comprehensive investigations offer a holistic understanding of how DSEK impacts patients' vision and well-being.

## Conclusions

In conclusion, our study's results show that, when properly corrected, DSEK significantly improves average visual acuity. The post-operative complications, while manageable, highlight the need for thorough patient and healthcare professional education regarding the nuances of this surgical approach. Descemet's stripping endothelial keratoplasty seems to be a promising replacement for conventional PK in patients with endothelial disorders. The advantages of DSEK point to its potential as a workable alternative, despite the need for more clarification of its long-term effects. It is important to recognize the study's limitations, particularly the small sample size and the brief post-follow-up period. It is crucial to conduct additional research in order to fully comprehend the complications and long-term effects of DSEK. It will be essential to increase sample sizes and lengthen the follow-up period in order to better understand the phenomenon.
